# Interaction and Stability of Nanobubbles and Prenucleation Calcium Clusters during Ultrasonic Treatment of Hard Water

**DOI:** 10.1021/acsomega.3c07305

**Published:** 2024-01-03

**Authors:** Eavan Fitzgerald, Anup Kumar, Sruthy Poulose, J. M. D. Coey

**Affiliations:** School of Physics, Trinity College Dublin, Dublin D02 PN40, Ireland

## Abstract

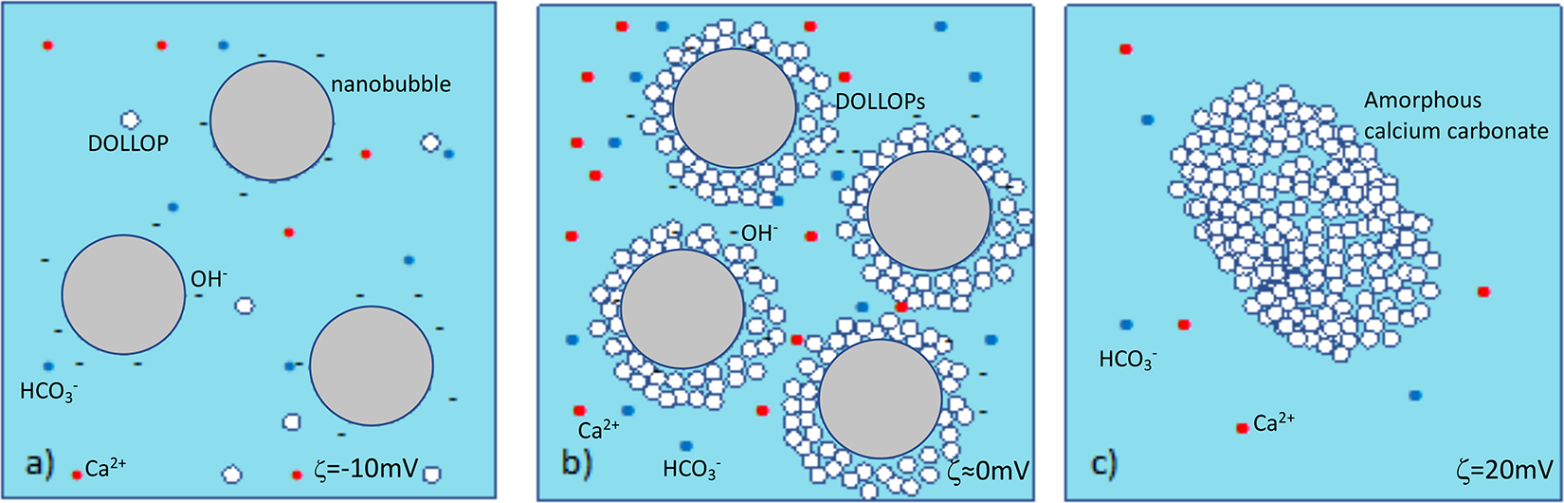

To investigate the stability of nanobubbles in natural hard water, a series of eight samples ranging in hardness from 0 to 332 mg/L CaCO_3_ were sonicated for periods of 5–45 min with an ultrasonic horn. Conductivity, temperature, ζ-potential, composition, and pH of the water were analyzed, together with the crystal structure of any calcium carbonate precipitate. Quasi-stable populations of bulk nanobubbles in Millipore and soft water are characterized by a ζ-potential of −35 to −20 mV, decaying over 60 h or more. After sonicating the hardest waters for about 10 min, they turn cloudy due to precipitation of amorphous calcium carbonate when the water temperature reaches 40 °C; the ζ-potential then jumps from −10 to +20 mV and remains positive for several days. From an analysis of the change of conductivity of the hard water before and after sonication, it is estimated that 37 ± 5% of calcium was not originally in solution but existed in nanoscale prenucleation clusters, which decorate the nanobubbles formed in the early stages of sonication. Heating and charge screening in the nanobubble colloid cause the decorated bubbles to collapse or disperse, leaving an amorphous precursor of aragonite. Sonicating the soft supernatant increases its conductivity and pH and restores the negative ζ-potential associated with bulk nanobubbles, but there is no further precipitation. Our study of the correlation between nanobubble production and calcium agglomeration spanning the hardness and composition ranges of natural waters shows that the sonication method for introducing nanobubbles is viable only for hard water if it is kept cold; the stability of the nanobubble colloid will be reduced in any case by the presence of dissolved calcium and magnesium.

## Introduction

The stability of bulk nanobubbles has come under increasing scrutiny in recent years as the list of potential applications grows ever longer and more diverse. Current uses range from horticulture^1^ and medicine^2^ to environmental applications,^3^ including mineral separation from tailings,^4^ wastewater treatment,^5^ and groundwater remediation.^6^ However, it has been a challenge to reach an agreed understanding of the factors that govern their formation and stability, in either ultrapure or natural water. The burgeoning applications in environmental and agricultural systems^7^ have to make use of local water, some of which will be inevitably hard or very hard. The current status of the field was recently surveyed in a special issue of Current Opinion in Colloid and Interface Science.^a^ Here, we report an investigation of quasi-stable nanobubble dispersions produced by acoustic cavitation in natural waters of widely different hardness. These are systems where gas-filled nanobubbles coexist with different populations of much smaller hydrated calcium carbonate nanoparticles. We want to understand how these two populations of incommensurate nano-objects interact on a submicroscopic scale^8,9^ and see if sonication is a useful method for reliably generating nanobubbles in natural waters of different hardness.

Water’s capacity for self-dissociation into its constituent ionic species distinguishes it as a universal amphiprotic solvent^10^ exhibiting acidic or basic properties depending on the solute and temperature
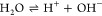
1

It is this duality and its high dielectric constant that make water such an effective solvent, exhibiting a wide variety of natural mineral compositions. A broad classification of water hardness is based on the concentration of dissolved cations, particularly Ca^2+^ and Mg^2+^. Within this classification, temporary hardness that can be removed by boiling is distinguished from permanent hardness that cannot.^11^ Permanent hardness is due to the presence of chloride or sulfate anions, forming complexes with dissolved cations that remain soluble at boiling temperatures. Temporary hardness is due to bicarbonate complexes whose solubility decreases with an increase in temperature, and they may be precipitated before the water boils. It leads to the formation of hard limescale on baths, cooking utensils, and hot water systems. Water at ambient temperature contains sufficient CO_2_ dissolved as carbonic acid to neutralize dissolved Ca^2+^ cations with HCO_3_^–^ anions, but the solubility of CO_2_ in water decreases with temperature according to the reaction
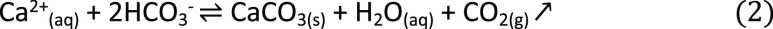
2

This release of CO_2_ promotes endothermic precipitation of calcium carbonate as the temperature approaches 80 °C,^12^ thereby softening the water by removing many of the Ca^2+^ ions from solution. The buildup of calcium carbonate as hard calcite limescale clogs the pipework and reduces the efficiency of boilers and heat exchangers. It is a significant economic problem.

**Figure 1:**
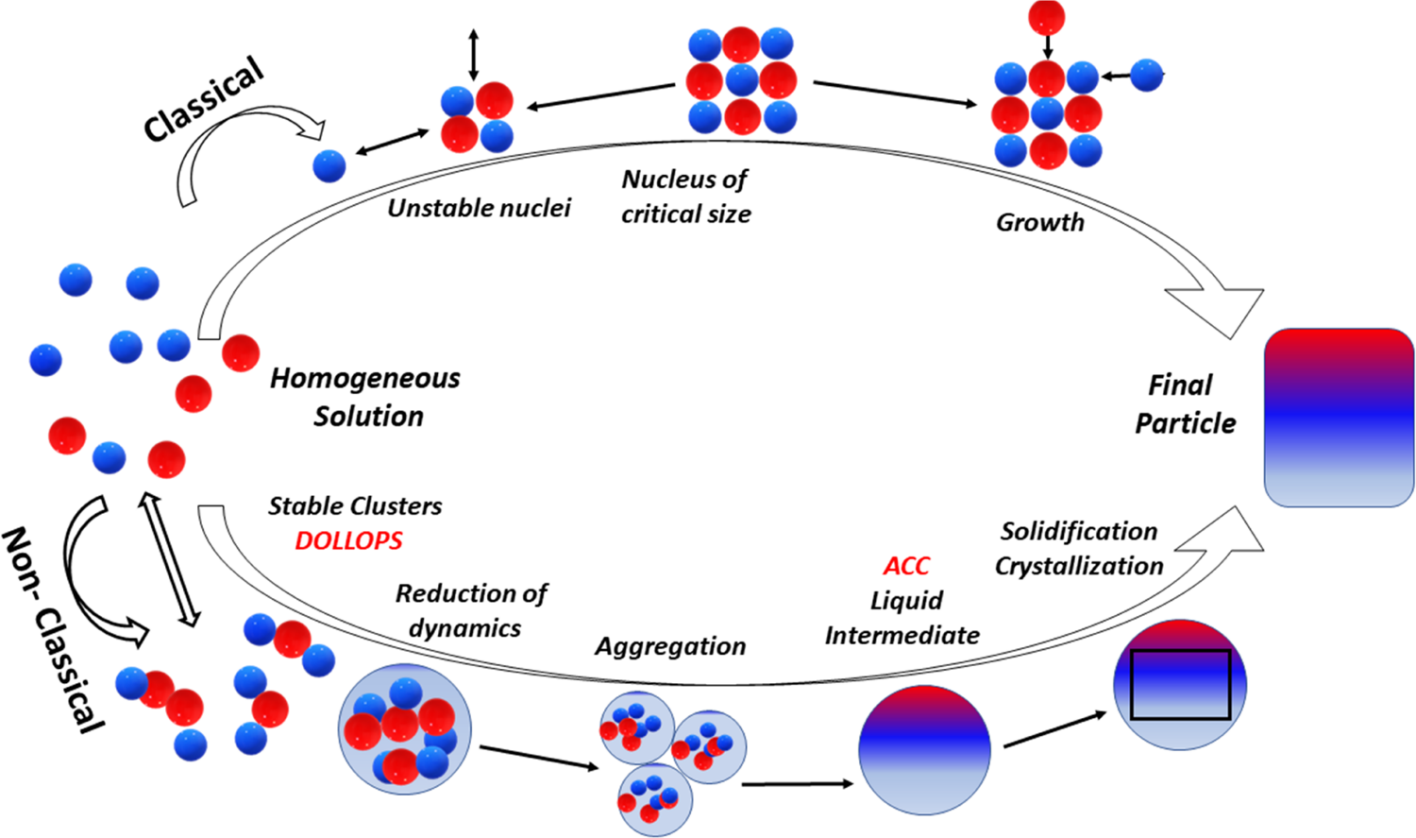
Classical and nonclassical nucleation pathways. [Adapted from ref (26) with permission of the American Journal of Science].

Efficient control of CaCO_3_ precipitation relies on a better understanding of the underlying mechanism of nucleation. The classical nucleation model of eq 2, illustrated in the top path of Figure 1, was defined by stochastic growth of crystalline nuclei that occurs only if a critical size is exceeded. The high enthalpic barrier to crystal formation means that spontaneous precipitation requires a supersaturated solution. An alternative mechanism, depicted along the bottom path in Figure 1, proceeds instead by aggregation of prenucleation clusters (PNCs)^13,14^ and has been found to compete with the classical model. These amorphous, hydrated polymeric nanoclusters of ions and counterions of approximate composition CaCO_3_.H_2_O, known also as DOLLOPs (dynamically ordered liquid-like oxyanion polymers),^15,16^ are thought to be present to some degree in any solution of CaCO_3_. Structurally they are dynamic folded polymeric chains with dimensions of about 2 nm,^13,15^ whose stability is a function of enthalpic conformational freedom, free energy of solvation, and Coulomb interaction energy. DOLLOPs helps to lower the enthalpic barrier to crystallization. Their aggregation precedes the formation of a dense liquid intermediate, having no formal phase boundary with the solvent,^17,18^ which then transforms to an amorphous form of calcium carbonate (ACC). Subsequent crystallization involves dehydration of the ACC with no large energy barriers.^19^ The kinetics of the transformation are not yet wholly understood as they involve variables including pH, solute, temperature, and ionic impurities.^20,21^ The existence of DOLLOPs is controversial, and some more recent studies have not found expected evidence of very small stable clusters in calcium bicarbonate solutions.^22−24^ The role of bicarbonate ions^25^ or superficial and structural water and impurities such as Mg^2+^ in ACC and its precursor(s) may be decisive for their stability.^21^

Another unknown is the enigmatic longevity of bulk nanobubbles. Although their existence has been established experimentally,^27−31^ a definitive theory of their stability has not been agreed. With stable radii less than 1 μm, they can survive for long periods in water, as Brownian motion negates the buoyancy force. However, according to the classical Young–Laplace theory, the excess surface pressure
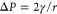
3

predicts their rapid collapse; Δ*P* exceeds 14 bar for a nanobubble of radius *r* = 100 nm in water with surface tension γ = 72 mN m^–1^. An outstanding question is how do the gas nanobubbles withstand the internal pressure without dissolving into the surrounding water? Seminal work^32^ discounted the existence of stationary nanobubbles outside of supersaturated solutions by considering a saturation-dependent diffusion model that predicts their disappearance within microseconds. Since then, bulk nanobubble research has established that their lifetime extends for periods ranging from hours to months, but explanations for stability of nanobubbles produced by ultrasonic cavitation^30,31,33−37,49^ and other methods^40,42−45,47−53^ continue to generate contoversy.

Two types of models have been advanced to account for their stability. One is based on a gas-impervious interface. Stabilization by surfactants or surface contamination is already employed to enhance the stability of ultrasound contrast agents^38,39^ where mechanical stress exerted by the adsorption of contaminants balances the Laplace pressure to create a stable equilibrium. Even in the case of partial coverage, a surfactant coating also functions as a diffusion barrier, enhancing nanobubble stability by establishing a dynamic equilibrium of gas flux at the interface.^40^ Attenuated total reflectance infrared spectroscopy has imaged hard hydrogen bonds at the bubble surface, similarly decreasing the diffusivity of the gas to prolong the nanobubbles’ life.^41^ The influence of surfactants on nanobubbles is the topic of a recent review.^42^

A different mechanism is required to account for the existence of nanobubble populations in pure water: here, models are based on charge stabilization. A countervailing Coulomb force arising from buildup of surface charge of either sign at the gas–liquid interface is critical for canceling the Laplace pressure and achieving a charge-stabilized nanobubble suspension.^27,43−45^ This idea is supported by a correlation between chemical additives and size distribution of the nanobubbles.^46^ In aqueous solutions, the negative surface charge is thought to be derived from preferential adsorption of hydroxyl anions, or other negatively charged species at the interface.^1,30,47−49^ The preference for hydroxyl anions is typically attributed to the difference in hydration enthalpies with hydronium cations, notionally supported by the increasing negativity of the ζ-potential at alkaline pH.^50,51^ Stability has also been attributed to the radial orientation of the water dipoles at the interface,^53−55^ charge transfer between water molecules,^56^ or a lower density of hydrogen bonds at the interface that limits the ionic adsorption capacity.^57,58^

The initial model of coulomb repulsion of charged nanobubbles proposed by Akulichev^59^ has since been expanded to account for the role of gas oversaturation at the interface, defining limits on the radius of a stable nanobubble^43,44^ in line with experimentally observed size ranges.^36^

In a model proposed by Zhang et al.,^44^ the size-dependent stability for the nanobubble is informed by a negative feedback mechanism derived by setting the derivative of the potential cost of formation of a charged bubble with respect to its radius *R* to zero such that
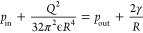
4

Equation 4 defines an equilibrium between a collapsing force on the right-hand side, a function of the Laplace and outer pressures *p*_out_, and an expansion force depending on the inner pressure *p*_in_ and surface charge density (*Q*: charge magnitude, ϵ: dielectric permeability) on the left.^44^ The inner pressure *p*_in_ is itself determined by the gas supersaturation ξ, which is a function of the concentration of the dissolved gas *C* in solution and the supersaturation concentration *c*_s_ such that
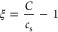
5

If the bubble shrinks, the electrostatic term dominates and the Coulombic force acts to restore the bubble to its equilibrium size (assuming *Q* remains approximately constant relative to a change in *R*). The theory proposed in ref (49) shows that the surface charge is naturally enriched by the shrinkage of a microbubble so that the Laplace force is balanced when the bubble reaches nanoscale dimensions. In practice, the surface potential is not resolvable, and so the ζ-potential, a measure of the electrokinetic potential at the shearing radius, is used as a proxy to characterize the stability of colloidal nanobubble dispersions. As distinct from the surface potential, it is strongly related to the particle mobility as it measures the potential difference between the dispersion medium and the layer of stationary fluid at the border of diffuse charge and adsorbed ions.^36,60^ The ζ-potential of a colloidal dispersion is a widely accepted indicator of its stability, due to a balance of intercolloid Coulomb repulsion and van der Waals attraction, treated by DLVO theory.^61,62^ This consideration is distinct from the stability of an individual nanobubble. The nanobubble colloid is regarded as stable when the ζ-potential is less (more negative) than −30 mV.^27,63^

A wide variety of different methods for generating stable nanobubble dispersions has been demonstrated.^8^ Aside from acoustic cavitation,^30,31,51,63−65^ which we adopt here, the methods include hydrodynamic cavitation,^66,67^ evolution of gas bubbles by electrolysis,^68,69^ direct injection of gas into solution,^70^ and application of magnetic^71^ or electric fields.^55^ As ultrasound is an indirect, energetic mode of nanobubble preparation, an investigation of the associated microscopic processes is needed to find out whether it is a viable method for use in natural waters of different hardness. This was the aim of our study.

## Materials and Methods

Widely available brand-name mineral waters Evian, Ballygowan, and Volvic were purchased locally, and three other natural water samples were prepared from Evian and Volvic mixtures. This approach was chosen to facilitate further studies by others on accessible, single-source water. Millipore deionized water of 18 MΩ cm was used as a reference, and soft Dublin mains tap water is included among our samples. Chemical analysis of all samples except Millipore water was conducted by the Public Analyst’s Laboratory, Dublin. The analysis of the seven natural waters used in this study, including three Evian/Volvic mixtures, are listed in Table 1, together with the reference Millipore deionized water. Based on total hardness as CaCO_3_, less than 100 mg/L is considered soft or moderately soft, and more than 200 mg/L is hard. In this way, Volvic is moderately soft, Ballygowan and Evian are very hard, and the 3:1, 1:1, and 1:3 mixtures are slightly hard, borderline hard, and hard, respectively.

**Table 1:** Mineral Composition in mg/L of Ultrasonically Treated Waters^72^^a^

	water sample					Volvic:Evian		
	Millipore	tap	Volvic	Ballygowan	Evian	3:1	1:1	1:3
pH	5.8	7.2	7.3	7.5	7.6	7.28	7.35	7.43
conductivity (at 20 °C)	<0.02	0.17	0.23	0.64 (0.58)	0.60 (0.54)	0.31	0.41	0.51
total hardness as CaCO_3_ (TDS)	0	59	70	316	332	136	201	267
total hardness as Ca		24	28	127	133	54	80	107
calcium		19.7	12	93.2	79.9	29	45	63
magnesium		1.8	8.2	20.1	27.5	13	18	23
sodium		7	12	15	7	10	9	8
potassium		–nd	6	3	1	5	3.5	2
chloride		12.7	14	24	10.6	15	13	12
sulfate		23.48	8.5	15	13.29	10	11	13

a Trace amounts are indicated by a dashed line. ζ-potential is measured in mV. Conductivity in mS/cm is standardized to 25 °C, with an error of ±0.01 mS/cm. Properties of three Volvic:Evian mixtures are included.

Glassware was subjected to three 15 min rounds of ultrasonic cleaning in acetone, ethanol, and isopropanol, followed by pure deionized water. Glassware was thoroughly rinsed directly before use with deionized water and then with the sample water.

Nanobubble dispersions were prepared by acoustic cavitation, following the method of Nirmalkar et al.^30,67^ A continuous 30 kHz ultrasound signal from a 100 W Hielscher UP100H ultrasonic generator was focused by a Ti-alloy horn with a 2 mm tip into a vial containing 25 or 3 mL of water (included to illustrate the temperature increase in a small volume) for periods of 5–45 min, while the temperature of the water was monitored with a thermocouple. 25 mL water samples were used for all experiments unless stated otherwise. Measurements were repeated to cover the range of total hardness as CaCO_3_ from 0 to 332 mg/L. In some cases, the vial was immersed in a 600 mL bath where the temperature was maintained below 20 °C by adding ice.

To control for secondary heating during sonication, 25 mL of each sample was heated on a hot plate without sonication. Once a threshold of 50 °C was reached, the temperature was maintained at 50–60 °C for 25 min.

The presence of nanobubbles was checked by Tyndall light scattering using a 532 nm green laser pointer. The stability of the nanobubble population was quantified through triplicate 100-fold measurements of the ζ-potential using a Malvern Zetasizer Pro instrument with a DTS1070 cell. The instrument uses electrophoretic light scattering (ELS) to measure zeta potential indirectly from the mobility of a charged nanoobject under the action of an applied electric field. A low nanobubble number density is assumed so that the optical properties of water (absorption and refractive index) are accurate approximations of the nanobubble material parameters for the quoted instrumental measurement range of 0.3 nm to 10 μm.

Nanobubble concentration and size distribution were determined in some cases by nanoparticle tracking analysis (NTA) using a Malvern NanoSight instrument, which tracks a flow of individual nanoobjects by laser light scattering or by a Particle Metrix ZetaView instrument that tracks their Brownian motion. Both methods yield similar concentrations, but the ZetaView is preferred as it avoids irreproducible oscillations in the reported size distribution.^73^
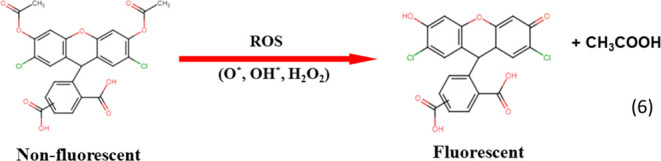


Conductivity and pH were monitored with a PC100 Cole Palmer meter calibrated fortnightly with a two-point system. Measurements of conductivity were also made in the Malvern Zetasizer on aliquots pipetted from the sample during the sonication period. A Vernier calcium electrode was also used to monitor the concentration of dissolved calcium at 10 min intervals during sonication. Dissolved oxygen in water samples was measured with an Oxyguard Polaris C meter. Reactive oxygen species such as OH*, O*, and H_2_O_2_ produced during sonication were measured in Millipore water as represented in eq 6 using a fluorescent dye [5(6)-carboxy-2′,7′-dichlorodihydrofluorescein diacetate from Merck]. These species are very short-lived and do not contribute to the colloidal stability of the nanobubbles.

Three samples labeled ″40 min ultrasound”, ″80 °C evaporation”, and “high-temperature evaporation” were prepared for XRD analysis of the dry precipitate obtained from hard water. Sedimentation of the coagulated precipitate occurred over several hours or days. The supernatant was then pipetted out, and the precipitate was dried at room temperature for the “40 min ultrasound” sample. The precipitate obtained on heating unsonicated hard water at 80° is the “80 °C evaporation” sample. The “high-temperature evaporation” sample was obtained by boiling unsonicated water to dryness. Precipitates were characterized using a Philips X’Pert PW 3040 Powder Diffractometer with Cu K_α_ radiation and λ = 0.1543 nm in the range 20° < 2θ *<* 60°. The data were fitted by using FullProf software to refine the aragonite and calcite lattice parameters.

Samples for analysis by scanning electron micrography using a Zeiss Ultra plus microscope were sonicated and then repeatedly filtered through 0.2 μm pore papers, which were dried in an oven at 70–80 °C.

A secondary sonication of the supernatant pipetted out after precipitation was conducted to analyze the role of the amorphous prenucleation clusters.

## Results

**Figure 2:**
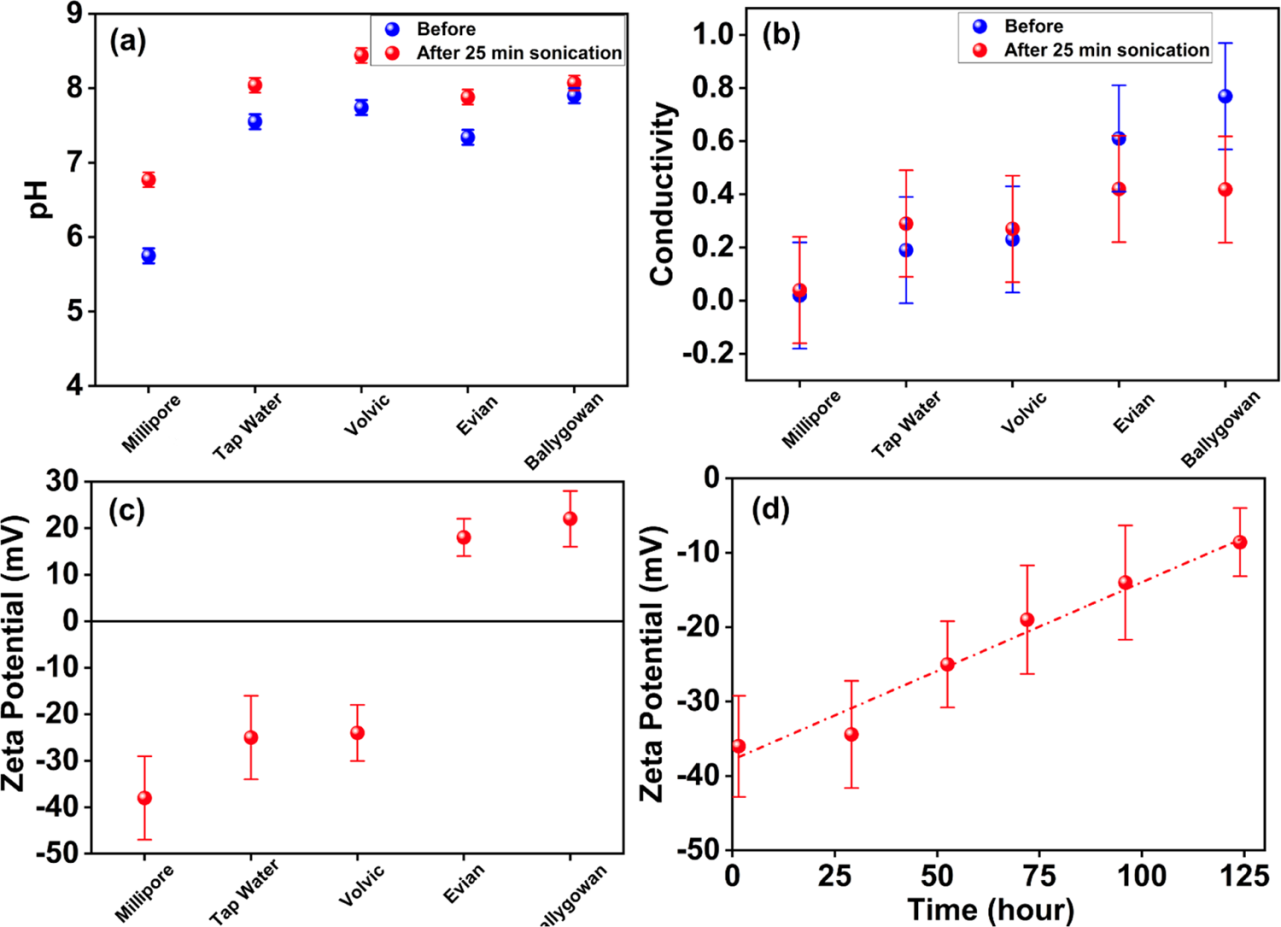
(a) pH and (b) conductivity before and after 25 min sonication and (c) ζ-potential after 25 min sonication. A charge-stabilized nanobubble suspension was formed in Millipore water, the time decay of which is shown in panel (d). Precipitation of CaCO_3_ by sonication of hard waters accounts for the significant decrease in conductivity shown in panel (b) relative to soft waters and positive ζ-potential in panel (c) postsonication.

The effect of a 25-min sonication on pH, conductivity, and ζ-potential of samples of the five reference waters is shown in Figure 2. Error bars are based on three (pH) or six measurements (conductivity and ζ-potential) for each data point. The pH increased by up to 1 unit after sonication, while conductivity increased slightly for soft waters but decreased for hard waters. Controlled heating experiments were used to distinguish the effect of direct heating from the effect of ultrasound.

The increase in pH, particularly in soft waters, may be attributable in part to the temperature-dependent solubility of CO_2_ gas in pure water. The reaction to form carbonic acid
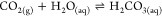
7

is impacted by the decrease of CO_2_ in solution at elevated temperature, which increases the pH. An analogous increase is observed by heating the samples directly. Moreover, the saturation with respect to calcium carbonate is inherently linked to this solubility, with precipitation occurring in a basic solution and at an elevated temperature. Notably, precipitation on sonication occurs for Evian and Ballygowan out of the five unmixed samples. Furthermore, Ballygowan water exhibited a negligible change in pH in the aftermath of sonication, but once the precipitate has been removed, the pH of the supernatant of Evian is increased by 0.3 and Ballygowan remains practically unchanged. This may in part be attributable to a fraction of H^+^ ions consumed in the formation of trace amounts of Ca(HCO_3_)_2_. Using the definition of pH = −log[H^+^], an increase in pH from 7.3 to 8.4 corresponds to the precipitation of a plausible 0.004 mg/L of calcium bicarbonate.

The ζ-potential after sonication is shown in Figure 2c. Except for tap water at −12 mV, the values before sonication were effectively undefined because almost no nanoparticles were observed in NTA. Heating without sonication at 50–60 °C for 25 min triggered only small changes |Δζ| of 0.5–8 mV.

**Figure 3:**
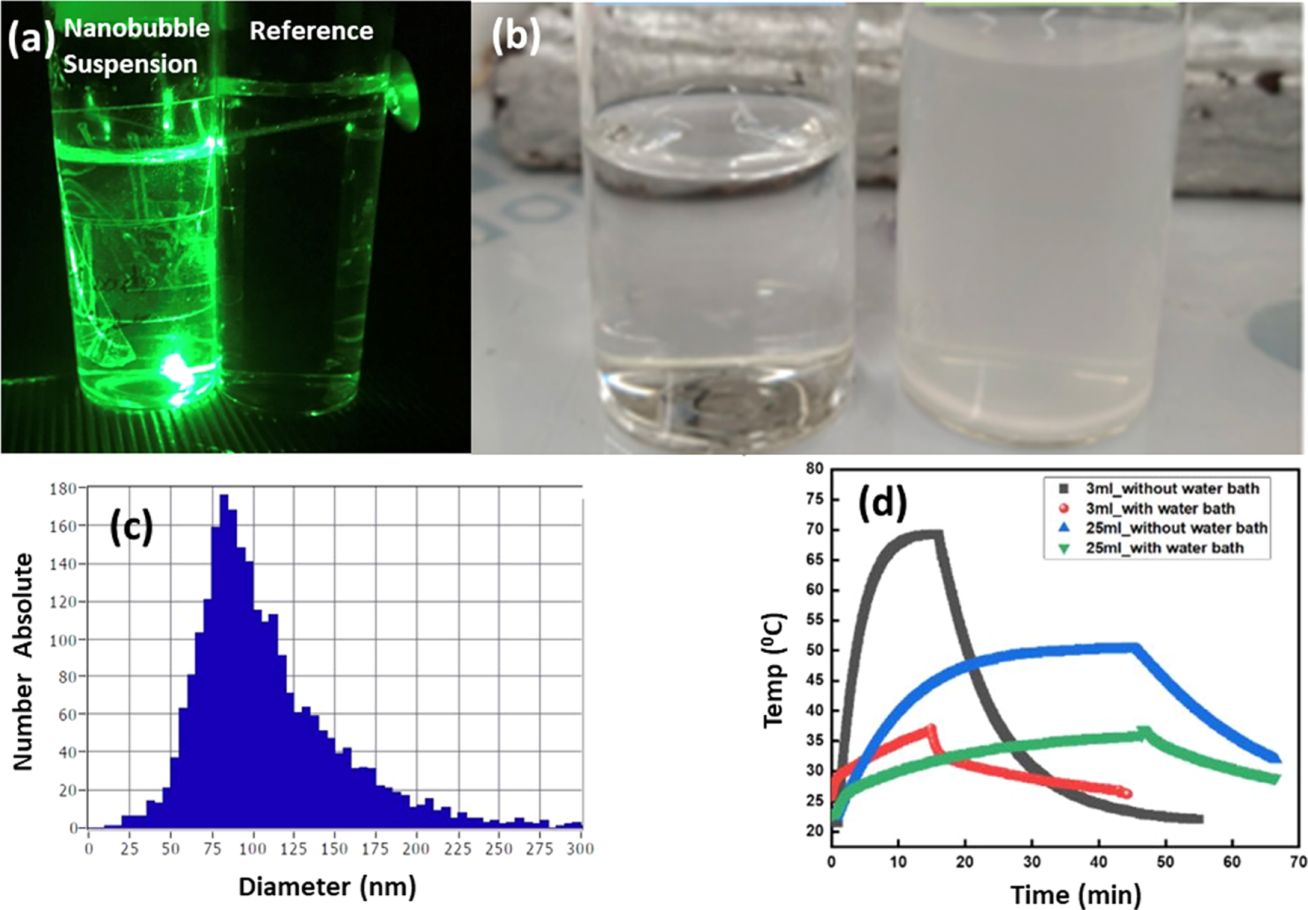
(a) Tyndall scattering of 532 nm green laser light by a nanobubble suspension in sonicated Millipore water; an unsonicated sample is shown on the right for reference. (b) Comparison of Millipore and Evian water after 25 min sonication; turbidity is due to the precipitation of calcium carbonate. (c) Population density of nanobubbles in sonicated Millipore water measured by laser particle tracking. (d) Monitoring of the temperature of Millipore water samples with sonication time. Ultrasound is switched off after 15 or 45 min.

A ζ-potential of −30 mV is a hallmark of a charge-stabilized nanobubble suspension.^63^ By this definition, only Millipore water qualifies, although soft Volvic and tap water, as well as the sonicated supernatant after removal of the precipitate, showed a decrease to −25 mV and exhibited characteristic Tyndall scattering (Figure 3a,b). The hard Evian mineral water only shows a Tyndall effect with a ζ-potential of −25 mV when sonicated in an ice-water bath that maintains the temperature below 20 °C. The stability of Millipore’s nanobubble population was monitored by the ζ-potential decay extending over 100 h, as shown in Figure 2d. ELS is inherently biased to register the larger bubbles,^28^ meaning that the decay may reflect shrinking bubble diameter as well as a decrease in number density.

The nanobubble size distribution was investigated by a nanoparticle tracking analysis. The laser NTA data shown in Figure 3c, where the number of nanobubbles is 3 × 10^8^/mL and the average diameter is 110 nm, are obtained with the ZetaView instrument. Most of the nanobubble volume is in the tail of the distribution, and the volume average particle diameter is 205 nm. (A consistently smaller density of 1.0 × 10^8^/mL and an average diameter of 130 nm with an oscillatory structure that differed from run to run was obtained from the NanoSight Instrument.) In a recent paper comparing methods used to determine nanobubble size distributions, the oscillatory structure is not seen when the dynamic light scattering (DLS) or interactive force analysis (IFA) methods are used.^73^ It seems to be an artifact of intense diffraction patterns produced by some of the nanobubbles flowing through the NanoSight field of view. Furthermore, the nanobubble diameters obtained by these two methods, especially DLS, are consistently larger than those found by NTA.

The concentration of reactive oxygen species (ROS) generated in sonicated Millipore water at 20 °C was 2.9 μM, as measured by fluorescence detection using the oxidative stress indicator “Carboxy-H2DCFDA”. Ultrasonication generates a micromolar concentration of ROS in solution that normally decays very fast, but the ROS are captured by the diacetate groups on the indicator and analyzed by fluorescence. A continuous supply of reactive oxygen species, predominantly OH*^74^ for applications such as wastewater decontamination^75^ or seed germination^74^ can be provided by collapse of nanobubbles in solution. Sonication of water at pH 7 (Figure 2a) increases the pH by about one unit, also the concentration of OH^–^ in solution, which serves to stabilize the nanobubbles with a layer of negative charge. For example, the concentration of OH^–^ at pH 7 of 10^–7^ M at 25 °C is sufficient to provide 16 hydroxide ions per nm^2^ of surface of 110 nm diameter nanobubbles, which is an order of magnitude greater than the charge needed to stabilize them.^76^

The high purity of Millipore leads us to expect that the ζ-potential that develops on sonication is a function of nanobubble formation alone. However, there is a question of whether the nano-objects seen in NTA, which are associated with the negative ζ-potential, might actually be a solid Ti-based particle contaminant shed by the horn during sonication.^33,35,36^ This shedding would be expected to occur in all samples. It was monitored versus sonication time by ICP-MS analysis. Only 150 ppb of Ti was present in Millipore water after 30 min sonication, which, if present as TiO_2_, corresponds to just 6% of the nanobubble volume measured by NTA. Moreover, other recognized methods of creating nanobubbles produce no titanium. Experiments where we used a method having no contact between water and metal yield NTA distributions and ζ-potential as a function of time and temperature that are similar to those found with ultrasonic cavitation using a titanium horn.

We are unsure what gas is in the bubbles, but the decrease with sonication time of dissolved oxygen in Millipore water is linear ith sonication s line; the observed decrease of order 1 μg/mL would be sufficient to fill 10^11^nanobubbles/mL with oxygen or air.

**Figure 4:**
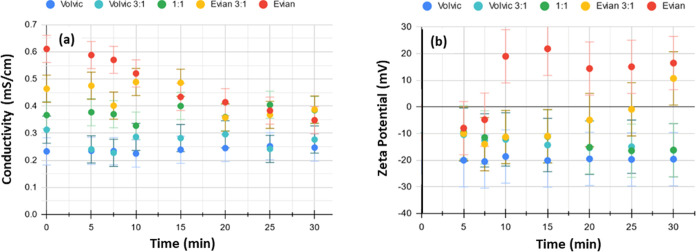
(a) In situ monitoring of conductivity and (b) ζ-potential of samples of increasing hardness during 30 min sonication.

The hard mineral waters Evian and Ballygowan behave quite differently from the soft waters after sonication; at first they show a ζ-potential of about −10 mV that then increases to a positive value of 20 mV, which is associated with the remarkable change in the appearance of the water shown in Figure 3b, due to precipitation of ACC once a threshold temperature of 40 °C is crossed. There is a reduction in dissolved calcium from 78.6 to 61.4 mg/L after extended 25 min sonication. The magnitude of the initial decrease is seen in Figure 4b to follow the degree of hardness, with the ζ-potential of the softer waters stabilizing at a negative value after approximately 15 min of sonication.

The temperature of the 25 mL samples always remains below 50 °C during sonication (Figure 3d), insufficient to cause precipitation in the absence of ultrasound. The change of appearance after sonication, when the hard water samples become cloudy and opaque, is attributed to flocculation of amorphous calcium carbonate (ACC), an aragonite precursor on the nonclassical nucleation pathway. NTA measurements on the samples with ζ-potential > +20 mV show nanoparticle populations of 3–4 × 10^7^ for Evian and Ballygowan with an average particle size of 80 nm for the ACC formed. Coagulation occurred over several days and was accelerated by heating. Precipitation induced by direct heating produced much larger particles, with no delay and no appearance of a positive ζ-potential, suggesting that the interaction between nanobubbles and prenucleation clusters in the early stages of sonication (where the ζ-potential is negative) affects the ionic configuration during initial formation of quasi-stable ACC, the intermediate product of DOLLOPs in the nonclassical pathway, Figure 1. Notably, there is a reduction in dissolved oxygen (DO) from 8.7 to 7.6 mg/L after extended 25 min sonication for Milipore water, but for Evian water it reduces only to 8.2 from 8.8 mg/L that may just be due to heat generated during sonication. A similar reduction from 8.8 to 8.3 mg/L in dissolved oxygen is observed for Evian water heated from room temperature to 50 °C in 30 min. This difference in reduced dissolved oxygen content for Milipore water (ΔDO = 1.1 mg/L) and Evian water (ΔDO = 0.6 mg/L) after sonication supports the proposed mechanism of nanobubble formation in Milipore water and nanobubble collapse during sonication for Evian water due to precipitation of ACC. Raiteri and Gale^19^ proposed that in some cases, it forms more readily than the crystalline phase by the disruption of the solvation layer due to the roughness of its surface. This is supported by the outcome affected by heat: lowering calcium carbonate solubility to promote flocculation thereby decreases the hydrated surface area, mediating the effect of the ACC surface.

Maintaining the temperature below 20 °C during sonication of hard water in the cooled water bath inhibits flocculation of calcium carbonate, and ζ remains negative, supporting the association of the positive ζ-potential with calcium carbonate precipitation. That the water hardness influences the ζ-potential of the dispersion is best shown by the results on Volvic:Evian mixtures in Figure 4. Only pure Evian and the 75% Evian mixture became cloudy during sonication, just when the ζ-potential becomes positive. For the softer mixtures and Volvic itself, the ζ-potential is stabilized quickly after the initial decrease. This corroborates the observation by Yasuda et al.^77^ that an equilibrium population of nanobubbles is established such that the ζ-potential becomes approximately constant irrespective of sonication time. Only heating by prolonged sonication decreases its magnitude. Moreover, the temperature of the solution in our case also approaches an equilibrium, never exceeding 50 °C for the 25 mL samples even after 80 min sonication. An exponential fit to the data of Figure 3d saturates at 51 °C.

Conductivity of the 75% Evian sample showed a decrease analogous to that of pure Evian, after 10 minutes of sonication whereas the conductivity of the 75% Volvic sample aligns with pure Volvic after 10 minutes of sonication. The conductivity of the 50% mixture appeared to vary within a narrow range during sonication but was effectively unchanged after 30 min, all of which is depicted in Figure 4.

Calcium ions in solution will tend to reduce the stability of the nanobubble colloid, even in the absence of DOLLOPs, since they can neutralize the negative surface charge on the nanobubbles.^78,79^ This was seen from the change of ζ-potential with calcium concentration in Millipore solutions of CaCl_2_, where there is no carbonate, and 400 mg/L was found to decrease the magnitude of the ζ-potential to −18 mV.

**Figure 5:**
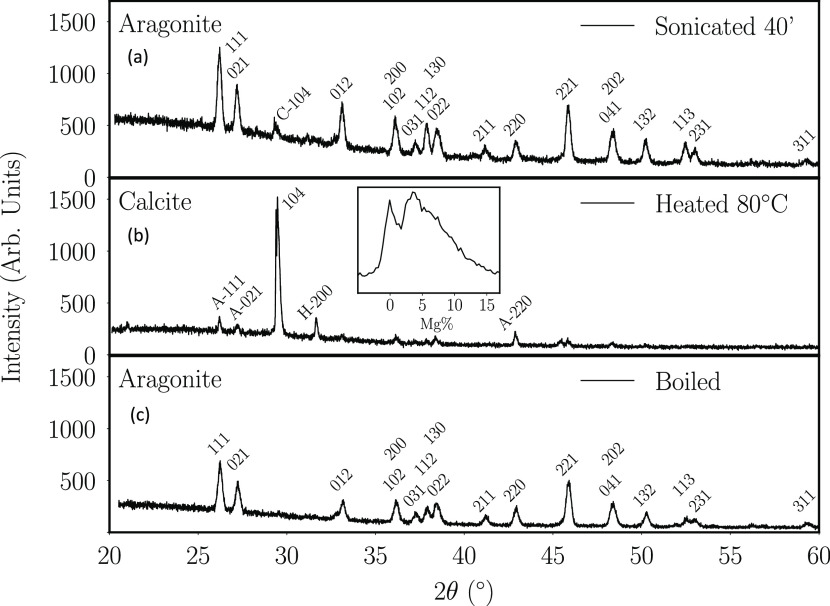
X-ray diffraction patterns of dried precipitate obtained from hard mineral water (Evian or Ballygowan). (a) Diagrams are for precipitates formed by 40 min sonication (top), (b) heating to 80 °C with no sonication (middle), and (c) boiling the water (bottom). The inset shows the 104 calcite reflection shifted by the distribution of Mg substitution for Ca.

**Figure 6:**
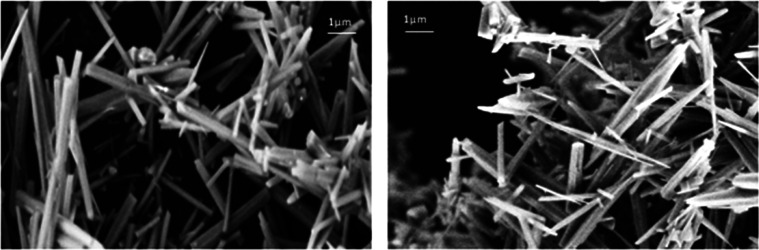
Scanning electron micrographs of precipitates from Evian (left) and Ballygowan (right) after sonication and dehydration at 80 °C. Needle-shaped aragonite crystals a few micrometers long are seen in both.

X-ray diffraction patterns in Figure 5 show that the precipitates are largely composed of calcium carbonates, with a minor amount of halite (H). Aragonite (A) is produced by ultrasound-induced precipitation^73,80^ or by boiling, whereas slow precipitation by heating to 80 °C produces calcite (C), the more stable polymorph. A minor amount of calcite (6%) is found in the 40 min sonicated aragonite and minor amounts of aragonite and halite in the 80 °C heated calcite. Scanning electron microscopy (SEM) images in Figure 6 illustrate the needle-shaped aragonite produced by sonicating hard water for 40 min. Aragonite is the dominant phase in the precipitate obtained by sonicating hard water.

Rietveld refinement of XRD patterns of aragonite and calcite precipitates gives the lattice parameters and cell volumes listed in Table 2, which are compared with those of the pure reference material. A greater Mg/Ca ratio associated with the smaller cell volume of carbonate precipitated from the hard water is consistent with the water analyses shown in Table 1.

**Table 2:** Lattice Parameters and Cell Volume of Orthorhombic Aragonite and Trigonal Calcite in the Precipitates from Ballygowan or Evian Hard Water; X-rays in Figure 5

	pure aragonite	pure calcite	40 min sonicated	heated to 80 °C^a^	boiled
*a* (Å)	5.016	4.99	4.960	4.99	4.962
*b* (Å)	8.035		7.970		7.972
*c* (Å)	5.812	17.06	5.744	17.05	5.742
*V* (Å^3^)	234.2	367.9	227.1		225.7

a 21% pure calcite, the remaining 79% with 5% Mg substitution.

## Discussion

The precipitation found during primary sonication of hard waters was not replicated in subsequent ultrasonic treatment of the supernatants; their pH is comparable but their conductivity, and therefore the total dissolved solids is lower: columns (ii) and (iv) in Table 3. Unlike the original hard water, a decrease in ζ-potential to −28 mV was observed on sonicating the supernatant; nanobubbles, but no ACC is produced. Also, unlike the original hard water, the conductivity, and therefore the quantity of free calcium, increases on sonicating the supernatant, due to the release of calcium ions from the DOLLOPs. The proportion of calcium bound in DOLLOPs must be at least 12% to account for the 0.08 ± 0.02 nS/cm increase in conductivity observed. The level of free calcium remaining in the ultrasonically softened supernatant, which now has a conductivity comparable to Volvic, is insufficient to nucleate precipitation (columns (i) and (iii)).

**Table 3:** pH, Conductivity, ζ-Potential of Hard Waters (i) Initially, (ii) after 25 min Sonication, (iii) the Supernatant, and (iv) as the Supernatant after 25 min Sonication

	Evian				Ballygowan			
	(i)	(ii)	(iii)	(iv)	(i)	(ii)	(iii)	(iv)
pH	7.3	7.8	7.6	7.8	7.6	7.8	7.5	7.8
σ (±0.05 mS/cm)	0.62	0.44	0. 26	0.32	0.78	0.42	0.28	0.38
ζ (±0.5 mV)	–1.6	18.0	–7.5	–27.5	–2.6	20.0	–5.3	–28.3

Crystallization of calcium carbonate from solution is reflected in the correlation between the decrease in conductivity during sonication and the initial water hardness, illustrated in Figure 4a, where all samples appear to converge to a final value of 0.20–0.40 mS/cm after 30 min. The total content of dissolved ionic solids (TDS) is generally related to σ, the conductivity at 25 °C, by the formula
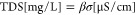
8

with the given units.^81^ The factor β depends on the ionic strength and nature of the ions in solution. Values can range from 0.45 to 0.75.^10^ Using the measurements of TDS and σ in Table 1 to estimate β, we find 0.52 and 0.53 for Evian and Ballygowan, respectively, which can be used to relate changes in conductivity (Δσ) to changes in TDS (ΔTDS). Ca^2+^ and CO_3_^2–^ ions bound in neutral DOLLOPs do not contribute to the electrical conductivity. Discrepancies between the value of ΔTDS and the mass of the extracted precipitate Δ*m*, both in mg/L, therefore allow us to estimate the fraction of calcium bound in DOLLOPs.

**Figure 7:**
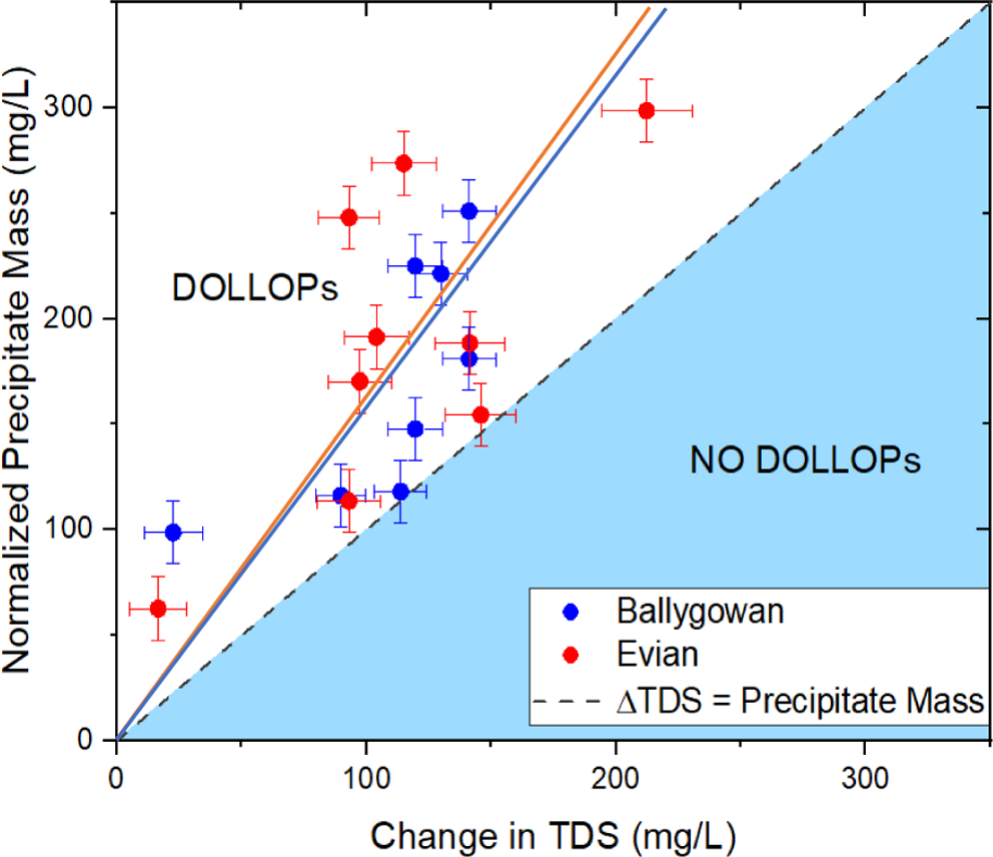
Plot of the change in total dissolved solids against the precipitate mass shows that the latter is consistently greater. Points in the white region indicate the presence of calcium carbonate in the water in the form of DOLLOPs that does not contribute to the conductivity. The slopes of the lines are 1.57 ± 0.10 for Evian and 1.53 ± 0.12 for Ballygowan.

Data for nine Evian samples and eight Ballygowan samples are plotted in Figure 7, where the black dashed line with slope 1 marks the threshold for the presence of DOLLOPs. The slopes of the red and blue fits to the data are practically identical. From the slopes, the proportion of calcium carbonate bound in DOLLOPs is 38% in Evian and 35% in Ballygowan, with an error of ±4%. These estimates are consistent with the lower limit of 12% deduced above from secondary sonication of the supernatant, and an experimental value of about 37% at pH 9.^15^ Remarkably, the Ca/Mg ratios of these two hard waters are rather different (Table 1), yet their total hardness and behavior in Figure 7 are similar.

Previous studies of particle–nanobubble interactions have been in the regime where the nanobubbles and nanoparticles (latex or gold) have comparable dimensions.^80,81^ Here, the dimensions of the solid and gaseous nanoobjects differ by a factor of at least 100, and the gas-filled nanobubbles have by far the larger mass. The nanobubble concentrations produced by ultrasonic cavitation are of order 10^8^/mL. Using the volume-averaged diameter of 205 nm, the volume fraction of bubbles is of order 1 ppm. The volume fraction of DOLLOPs is 6 ppm in soft water, assuming a CaCO_3_ concentration of 30 mg/L and 37% of the calcium present in DOLLOPs with a density of 2 g/mL. In hard water, the fraction will be 10 times greater. The volume of DOLLOPs appears to be greatly in excess of the volume of nanobubbles, but if the nanobubble diameter and population are larger, as suggested by DLS measurements,^73^ they might be comparable. Uncharged DOLLOPs are expected to be attracted to the nanobubbles by van der Waals interactions and envelop them, reducing the negative charge at the slipping surface and changing the ζ-potential of softer water, which ranges from −35 to −15 mV, provided their concentration is less than about 30 mg/L (1:1 Evian:Volvic mixture in Figure 4). However, the nanobubbles become unstable in harder water where the ζ-potential is initially about −10 mV, corresponding to a weakly charge-stabilized nanobubble colloid where the Coulomb repulsion is screened by the dissolved charge. The Debye–Huckel screening length λ_D_ = √{*k*_B_*T*ε/2e^2^*n*^*∞*^}, where ε is the dielectric permeability of the water and *n*^*∞*^ is the ionic density in the water far from the charged nanobubbles, is 70 nm or more in soft water and 30 nm in hard water. The gradual collapse of ζ-potential and its increase to +20 mV after 10 min in hard waters when the temperature has increased to 40 °C suggests that the sheathed nanobubbles become unstable, tending to coagulate and disperse or collapse, leaving behind extended particles of amorphous calcium carbonate, the precursor of aragonite. The positive ζ-potential coincident with the onset of precipitation of aragonite is consistent with previous work,^82^ although the ζ-potential of well-crystallized calcium carbonate is usually negative.^83,84^

All of this can br avoided when sonicating Ballygowan or Evian (they are ∼2 mM solutions of divalent ions) in an ice-water bath where they develop ζ-potentials of −25 mV without precipitation. There was no precipitation either when sonicating permanently hard CaCl_2_ or MgCl_2_ aqueous solutions where nanobubbles form at a less negative ζ-potential than in pure water, −15 to −20 mV in 3.6 mM solutions. Divalent cations displace the negatively charged species at the nanobubble surface,^61^ but anionic surfactants may be used to counteract the influence of cations near the nanobubble surface.^43^

**Figure 8:**
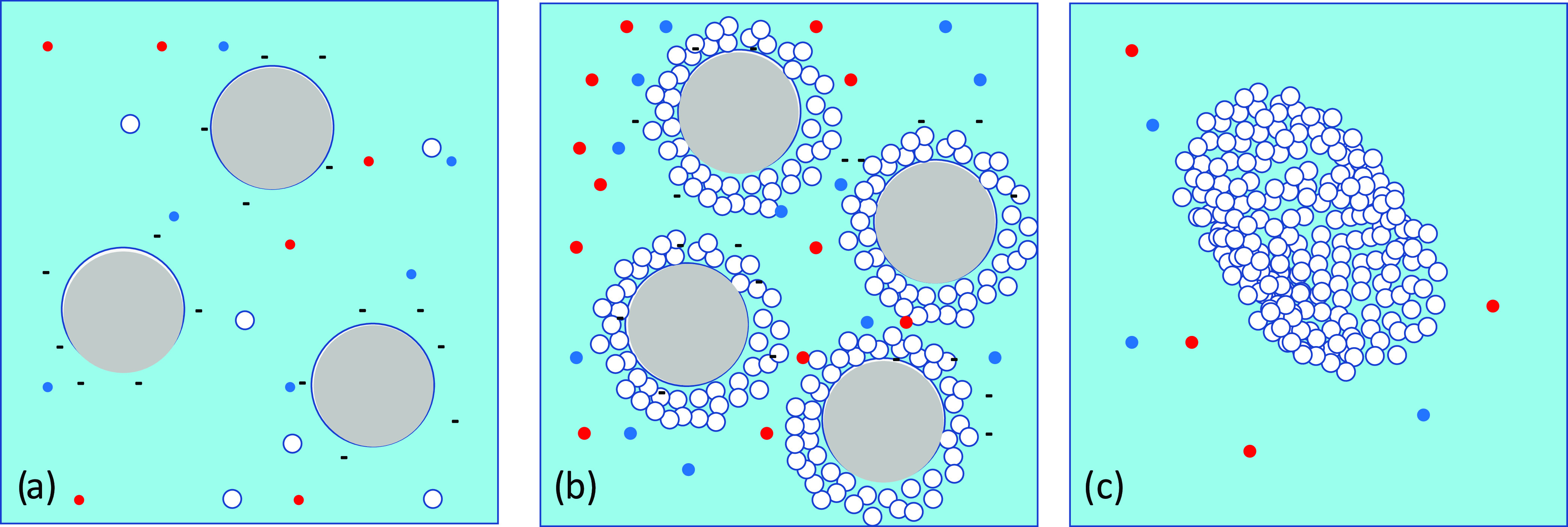
(a) Schematic nanostructure of sonicated soft water showing a charge-stabilized suspension of negatively charged nanobubbles (large gray circles) with some much smaller DOLLOPs (white circles) and Ca^2+^ and HCO_3_^–^ ions (red and blue dots) in solution. Here, ζ ≈ −30 mV. (b) Nanobubbles in hard water decorated with layers of DOLLOPs. The suspension is now weakly charge-stabilized by the reduced negative charge and screening of the internanobubble Coulomb repulsion by the greater concentration of Ca^2+^ ions. Here, ζ ≈ −10 mV. (c) Aggregation of the DOLLOPs on further sonication (*T* ≈ 40 °C) to form amorphous calcium carbonate, the precursor of crystalline aragonite. Here, ζ ≈ +20 mV. The nanobubbles coarsen and float away.

The process described for the precipitation of ACC and elimination of nanobubbles is illustrated schematically in Figure 8.

## Conclusions

Ultrasonic treatment is an effective method for producing quasi-stable suspensions of nanobubbles in soft water at pH 7–8, indicated by a negative ζ-potential between −35 and −20 mV. The volume of nanobubbles is significantly greater than could be explained by any titanium-based nanoparticles shed by the ultrasonic horn.

We have studied how populations of quite different sorts and sizes of soft matter―nanobubbles and polymeric prenucleation clusters―form and interact during sonication of hard water, proposing the scheme summarized in Figure 8. Nanobubble formation in early-stage sonication of hard water, indicated by a reduced ζ-potential of about −10 mV is attributed to layers of the uncharged 2 nm prenucleation clusters of calcium carbonate known as DOLLOPs that envelope the nanobubbles and reduce the concentration of hydrated OH^–^ ions or other available negatively charged species near their surface. At −10 mV, the nanobubble colloid is only weakly charge-stabilized. Further sonication and heating lead to an increase of ζ-potential, collapse of the nanobubbles, and aggregation of the associated DOLLOPs as amorphous calcium carbonate. While flocculation occurs spontaneously over time as the ζ-potential decays, the formation of a crystalline polymorph is accelerated by heating. The precipitate is largely aragonite for ultrasound-induced precipitation or boiling and calcite for precipitation at elevated temperature without ultrasound. Carbonate precipitation can be avoided entirely and the nanobubble population in hard water stabilized by keeping the water cold during sonication, although the stability of the nanobubble colloid will be reduced by the presence of Ca^2+^ and Mg^2+^ ions in solution.

Sonicating the soft supernatant leads to no further precipitation, but there is an increase in the conductivity and generation of nanobubbles. This, coupled with consistent discrepancies between the normalized mass of the precipitate and the change in total dissolved solids deduced from conductivity before and after initial sonication confirms the presence of some of the calcium as DOLLOPs in hard mineral waters, which favor a nonclassical nucleation pathway via amorphous calcium carbonate. The fraction of calcium held in these prenucleation clusters, which seed nucleation by coagulating during ultrasonic treatment, is estimated to be 37 ± 5%. The remainder is dissolved in ionic solution.

The strong local heating that occurs during ultrasonic cavitation probably explains why precipitation of calcium carbonate from hard water occurs at water temperatures that are about 40 °C lower than normal. In order to benefit from nanobubble generation of reactive oxygen species on collapse in hard water, it will be necessary to cool the water while sonicating or else use a less energetic method to produce them.

## Footnotes

a Nanobubbles and Nanodroplets, Edited by V. Craig, M. P. Kraft and T. Zemb, doi.org.10.1016/c.cocis.2021.101470.
